# Subcellular storage and release mode of the novel ^18^F-labeled sympathetic nerve PET tracer LMI1195

**DOI:** 10.1186/s13550-018-0365-9

**Published:** 2018-02-06

**Authors:** Xinyu Chen, Rudolf A. Werner, Constantin Lapa, Naoko Nose, Mitsuru Hirano, Mehrbod S. Javadi, Simon Robinson, Takahiro Higuchi

**Affiliations:** 1 Department of Nuclear Medicine, University Hospital Würzburg, Oberdürrbacher Strasse 6, 97080 Würzburg, Germany; 2 Comprehensive Heart Failure Center, University Hospital Würzburg, Würzburg, Germany; 3 The Russell H. Morgan Department of Radiology and Radiological Science, Division of Nuclear Medicine and Molecular Imaging, Johns Hopkins University School of Medicine, Baltimore, MD USA; 4 Department of Bio Medical Imaging, National Cardiovascular and Cerebral Research Center, Suita, Osaka Japan; 5 Lantheus Medical Imaging, North Billerica, MA USA

**Keywords:** Heart failure, Sympathetic nervous system, Storage vesicle turnover, Positron emission tomography, ^18^F-LMI1195, Phaeochromocytoma

## Abstract

**Background:**

^18^F-*N*-[3-bromo-4-(3-fluoro-propoxy)-benzyl]-guanidine (^18^F-LMI1195) is a new class of PET tracer designed for sympathetic nervous imaging of the heart. The favorable image quality with high and specific neural uptake has been previously demonstrated in animals and humans, but intracellular behavior is not yet fully understood. The aim of the present study is to verify whether it is taken up in storage vesicles and released in company with vesicle turnover.

**Results:**

Both vesicle-rich (PC12) and vesicle-poor (SK-N-SH) norepinephrine-expressing cell lines were used for in vitro tracer uptake studies. After 2 h of ^18^F-LMI1195 preloading into both cell lines, effects of stimulants for storage vesicle turnover (high concentration KCl (100 mM) or reserpine treatment) were measured at 10, 20, and 30 min. ^131^I-*meta*-iodobenzylguanidine (^131^I-MIBG) served as a reference. Both high concentration KCl and reserpine enhanced ^18^F-LMI1195 washout from PC12 cells, while tracer retention remained stable in the SK-N-SH cells. After 30 min of treatment, ^18^F-LMI1195 releasing index (percentage of tracer released from cells) from vesicle-rich PC12 cells achieved significant differences compared to cells without treatment condition. In contrast, such effect could not be observed using vesicle-poor SK-N-SH cell lines. Similar tracer kinetics after KCl or reserpine treatment were also observed using ^131^I-MIBG. In case of KCl exposure, Ca^2^+-free buffer with the calcium chelator, ethylenediaminetetracetic acid (EDTA), could suppress the tracer washout from PC12 cells. This finding is consistent with the tracer release being mediated by Ca^2^+ influx resulting from membrane depolarization.

**Conclusions:**

Analogous to ^131^I-MIBG, the current in vitro tracer uptake study confirmed that ^18^F-LMI1195 is also stored in vesicles in PC12 cells and released along with vesicle turnover. Understanding the basic kinetics of ^18^F-LMI1195 at a subcellular level is important for the design of clinical imaging protocols and imaging interpretation.

**Electronic supplementary material:**

The online version of this article (10.1186/s13550-018-0365-9) contains supplementary material, which is available to authorized users.

## Background

The single-photon emission computed tomography (SPECT) tracer ^123^I-*meta*-iodobenzylguanidine (MIBG) targeting norepinephrine transporter (NET) is currently the most widely used clinical tracer for sympathetic nervous imaging with well-established protocols and mature guidelines based on the results achieved from several clinical trials [1, 2]. However, positron emission tomography (PET) tracers show beneficial properties compared with SPECT tracers due to the development of imaging technology over the last couple of decades. PET provides superior sensitivity and improved temporal and spatial resolution along with the possibilities of regional cardiac imaging and kinetic studies for quantification [3]. Among the PET tracers that are currently available for NET imaging, a new class of ^18^F-labeled agents has drawn attention because of their longer half-life of fluorine-18 (110 min) over carbon-11 (20 min). Thereby, these ^18^F-labeled tracers provide a unique opportunity to further enhance the development and application of PET imaging in terms of reduction of the financial burden of hospitals, flexible novel tracer design, and labeling procedure with improved stabilities [4].

Currently, a couple of ^18^F-labeled tracers targeting the NET are available: *N*-[3-bromo-4-(3-^18^F-fluoropropoxy]-benzyl]-guanidine (^18^F-LMI1195) is designed for assessment of sympathetic innervation of the heart and has successfully passed through phase I clinical trial, which confirmed its tolerance in human subjects along with favorable biodistribution for cardiac imaging [5]. [^18^F]4-fluoro-3-hydroxyphenethylguanidine ([^18^F]4F-MHPG) and its isomer [^18^F]3-fluoro-4-hydroxyphenethylguanidine ([^18^F]3F-PHPG) have also been developed in order to counteract the perfusion dependence compared to previous NET tracers [6]. The first-in-human studies of both tracers showed clear and long-term cardiac retention [7].

All the abovementioned tracers share a similar structure (benzyl/phenethyl guanidine) as MIBG and therefore represent comparable properties. Among them, ^18^F-LMI1195 has so far caught most of the attentions from researchers due to its easy and high-yield labeling procedure that is convenient and eligible for commercial preparation and application [8, 9]. Similar to MIBG, ^18^F-LMI1195 is resistant to metabolism by monoamine oxidase [5, 10]. In a head-to-head comparison of ^18^F-LMI1195 with ^123^I-MIBG in isolated perfused rabbit hearts, tracer washout after vesicle turnover was accelerated by electrical field stimulation. Additionally, our group has also demonstrated that the retention of ^18^F-LMI1195 is resistant to desipramine chase (desipramine added after tracer delivery), which emphasizes its potential of mimicking the physiological norepinephrine turnover [11].

Nonetheless, although our former investigation on isolated rabbit rat heart has proved the accumulation of ^18^F-LMI1195 in nerve terminals, it was not sufficient enough to come to the conclusion that it was taken up into the vesicles. In a previous study, by using potassium chloride (KCl) and reserpine stimulation, the difference between extravesicular retention and granular storage of MIBG was clearly demonstrated in PC12 (vesicle-poorvesicle-rich) and SK-N-SH (vesicle-richvesicle-poor) cell lines [12]. Therefore, in order to gain further insights and clarify the kinetics of ^18^F-LMI1195 at a subcellular level, we aimed to compare it with its SPECT counterpart ^131^I-MIBG in both cell lines, as mentioned above, with regard to KCl or reserpine-induced tracer depletion mechanisms. High concentration of KCl has been applied as a simulant of electrical field stimulation that enhances cardiac LMI1195 washout significantly in the isolated rabbit heart [11]. Reserpine can also deplete catecholamines (in this case, ^18^F-LMI1195 that presumably mimics neurotransmitter) from storage vesicles [13]. By accomplishing this study, it might be possible and prove necessary to investigate the likely drug-tracer competition and to compare the different tracer uptake behavior and mechanism details. The conclusions achieved from the results will serve as a useful guidance for future clinical assessment.

## Methods

### Radiopharmaceuticals

^18^F-LMI1195 was synthesized and purified as described in the literature [8]. The radiochemical purity of the final product was greater than 95% with a specific radioactivity more than 10 GBq/μmol. ^131^I-MIBG was purchased from GE Healthcare (Freiburg im Breisgau, Germany) and used within 2 h after calibration time. ^131^I-MIBG was chosen instead of ^123^I-MIBG due to its relative longer half-life, which is convenient for research purposes and financial reasons.

### Cell culture

Both PC12 cells (adrenal gland pheochromocytoma from rat) and SK-N-SH cells (human neural cells from Caucasian neuroblastoma) were purchased from Sigma-Aldrich (Sigma-Aldrich Chemie GmbH, Munich, Germany) and were cultivated at 37 °C and 5% CO_2_. PC12 cells were grown in a Roswell Park Memorial Institute medium with 2 mM glutamine, 5% fetal bovine serum (FBS), and 10% horse serum. SK-N-SH cells were grown in MEM medium with 2 mM glutamine and 10% FBS. The cells were first grown in 75-cm^2^ flasks with type IV collagen coating, in which the cells would be adherent. One day prior to release assay, they were transferred to 12-well plates with 1 mL volume per well and 2 × 10^5^/mL density.

### Release assay

#### High concentration KCl-induced tracer release

Firstly, cells were incubated with high concentration KCl (100 mM) for 10, 20, and 30 min. The total protein concentrations after incubation were compared with control groups using only HEPES buffered saline (HBS) buffer (cf. Additional file 1) to insure the cell viability. No statistical difference could be concluded from these two groups. Therefore, this incubation condition was used for the following high concentration KCl induction study.

The culture medium was removed and the cells were washed with the medium. Cells were first incubated with radiotracers in a solution containing both ^18^F-LMI1195 (300 kBq) and ^131^I-MIBG (37 kBq) at 37 °C for 120 min. After incubation, the cells were washed twice with warmed HBS buffer. One milliliter of HBS buffer was added again followed with 5 min incubation before removal. Then, cells were treated with HBS (with or without Ca^2+^) or 100 mM high KCl buffer (with or without Ca^2+^) for 10, 20, and 30 min. After the treatment, the buffer was collected as the extracellular fraction. Cells were washed twice with ice-cold phosphate buffered saline (PBS) and solubilized in 0.1 N NaOH. Radioactivity in each sample was measured using a gamma counter using differential energy windows (± 20%) for ^18^F and ^131^I (FH412; Frieseke & Höpfner, Erlangen, Germany).

#### Reserpine-induced tracer release

Tracer loadings were performed in analogy to the abovementioned KCl study. Cells were incubated with radiotracers in a solution containing both ^18^F-LMI1195 (300 kBq) and ^131^I-MIBG (37 kBq) at 37 °C for 120 min. After the incubation period, cells were washed twice with warmed medium, followed by 5 min incubation with medium. Afterwards, cells were treated with a reserpine solution at final concentrations of 50 nM for PC12 cells and 5 μM for SK-N-SH cells for 10, 20, or 30 min, respectively, because it is known that PC12 cells are sensitive to reserpine-induced depletion, whereas a much higher concentration of reserpine is applied to SK-N-SH cells because of its dramatically lower storage capacity [13]. The incubation buffer was collected followed by double washing with ice-cold PBS. The cells were then solubilized in 0.1 N NaOH, and the cell lysate was collected. Radioactivity of each sample was measured using a gamma counter. Nonspecific uptake was measured in the presence of 10 μM of the selective NET inhibitor desipramine, and specific uptake was calculated by subtracting nonspecific radioactivity from total counts.

#### Retention index calculation

To quantify tracer release from cells, a retention index was calculated as

$$ \mathrm{Retention}\ \mathrm{index}\ \left(\%\right)=100\times \left(1-\mathrm{release}\ \mathrm{counts}/\mathrm{total}\ \mathrm{counts}\right), $$

in which release counts are defined as counts bound to extracellular buffer after release stimulation. Total counts are the counts bound to cell lysate after the tracer uptake period (including the washing process). To exclude non-specific binding or uptake (which does not contribute to release after vesicular turnover), non-specific uptake was determined in the presence of 10 μM desipramine and subtracted from total uptake.

### Statistics

All experimental data are presented as mean ± SD, with individual numbers measured in triplicate in experiments performed on 2–3 separate days. Statistical comparison of uptake/release ratios between two groups was performed by Student’s *t* test, where *p* values of less than 0.05 were considered statistically significant. Data were analyzed by analysis of variance (ANOVA) when multiple groups were compared. Statistical analysis was performed on GraphPad Prism (GraphPad Software, Inc., La Jolla, USA).

## Results

### High concentration KCl-induced tracer depletion

**Fig. 1:**
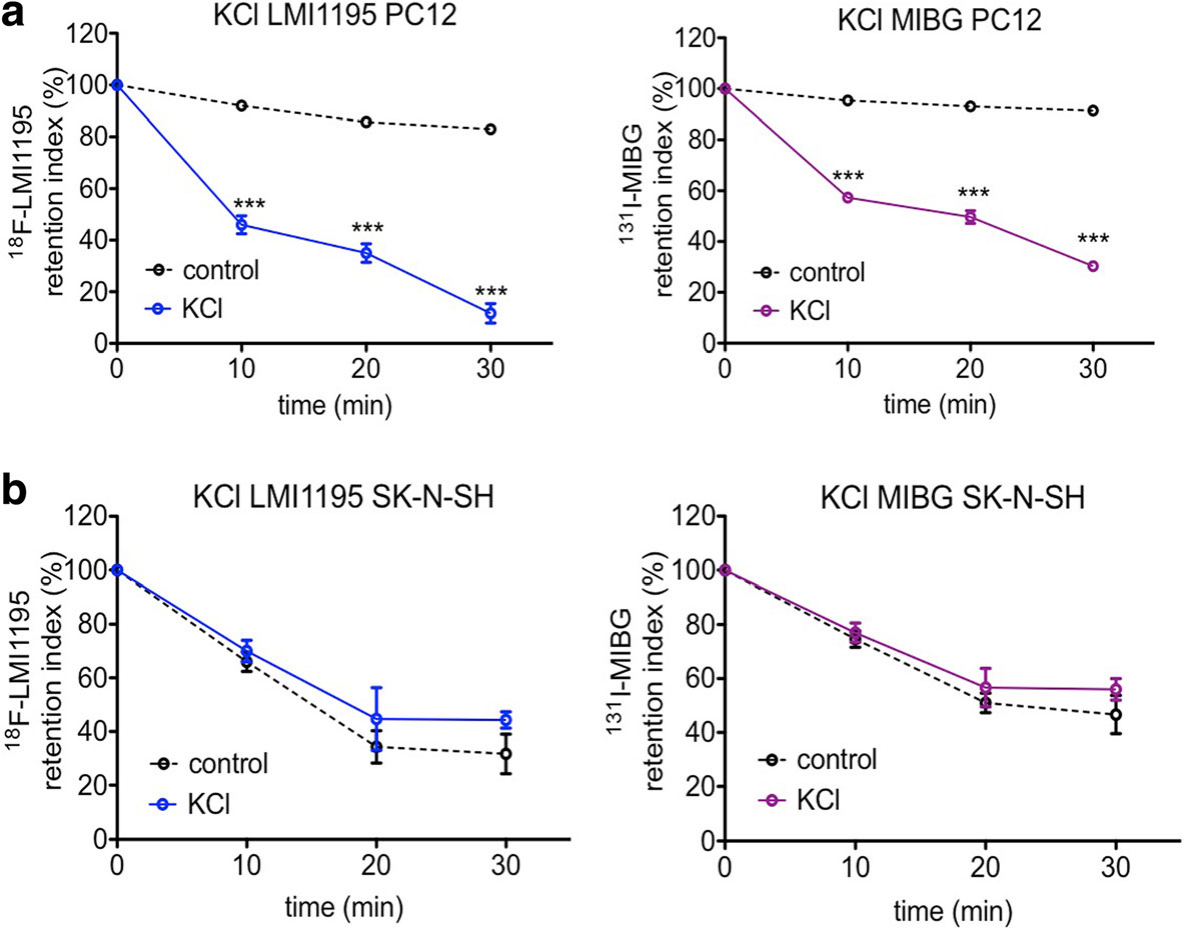
Time course of tracer retention index after the stimulation with high concentration KCl buffer. Both ^18^F-LMI1195 and ^131^I-MIBG were induced to be released from (vesicle-rich) PC12 cells after high concentration KCl buffer treatment (**a** *n* = 3 at each time point of both groups), whereas no such effect could be observed in (vesicle-poor) SK-N-SH cells (**b** *n* = 3 at each time point of both groups). ***p < 0.001 vs. control group at the same time point; all data points presented as mean ± SD

Treatment of PC12 cells with high concentration KCl buffer induced robust tracer depletion of both ^18^F-LMI1195 and ^131^I-MIBG in a time-dependent manner, leading to 88 ± 4% of ^18^F-LMI1195 and 70 ± 2% of ^131^I-MIBG total uptake released from cells (*p* < 0.001, vs. untreated controls, Fig. 1a). In contrast, KCl did not produce an obvious release of either ^18^F-LMI1195 or ^131^I-MIBG in SK-N-SH cells and demonstrated similar retention indices as controls (Fig. 1b).

**Fig. 2:**
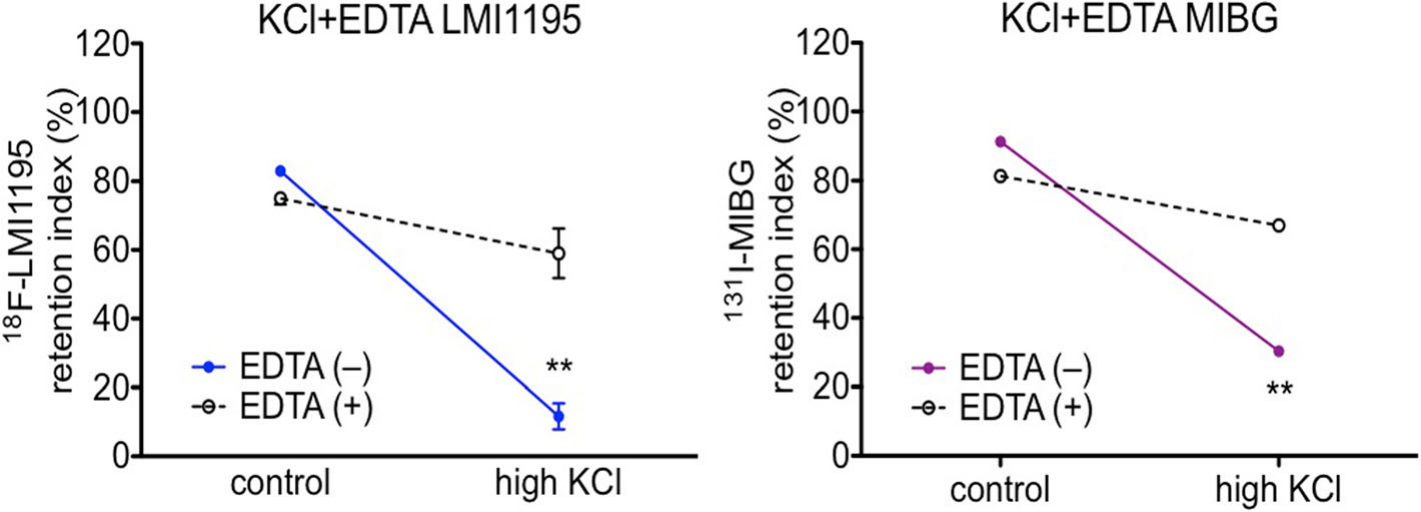
Comparison of both tracers in PC12 cells treated with 100 mM KCl in the presence (+) or absence (−) of EDTA. *Y*-axis represents the difference of release index, i.e., counts over control after 30 min of treatment with high concentration KCl buffer. Both ^18^F-LMI1195 (*n* = 3 of each testing group) and ^131^I-MIBG (*n* = 3 of each testing group) were released from PC12 cells after treatment with high concentration KCl in the absence of EDTA, whereas the effect was mitigated in the presence of EDTA. **p < 0.005 vs. EDTA (+) group at the same condition; all data points presented as mean ± SD

Tracer depletion in PC12 cells 30 min after treatment with high concentration KCl could be overturned by using Ca^2+^-free buffer containing ethylenediaminetetraacetic acid (EDTA) (Fig. 2). The release index (as percentage of tracer released from cells after certain treatment) of ^18^F-LMI1195 decreased from 71 ± 4% (100 mM KCl) to 16 ± 7% (100 mM KCl + EDTA). By investigating ^131^I-MIBG, the same tendency with a decrease from 61 ± 2% (100 mM KCl) to 15 ± 4% (100 mM KCl + EDTA) was observed (*p* < 0.005, respectively).

**Fig. 3:**
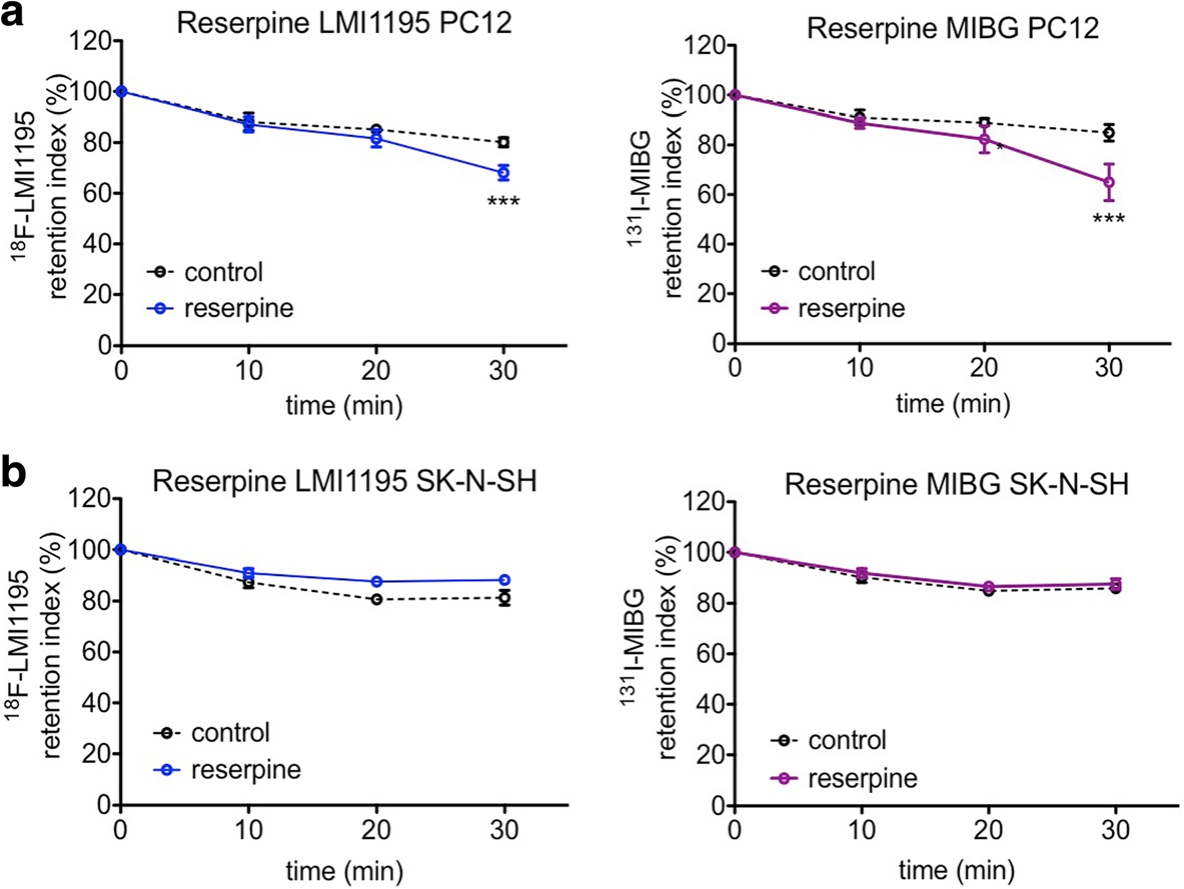
Time course of tracer retention index after the stimulation with reserpine. Both tracers are induced to be released from PC12 cells after reserpine treatment (**a** *n* = 6 at each time point of both groups), whereas such effect could not be observed in SK-N-SH cells (**b** *n* = 6 at each time point of both groups). ***p < 0.001 vs. control group at the same time point; all data points presented as mean ± SD

**Fig. 4:**
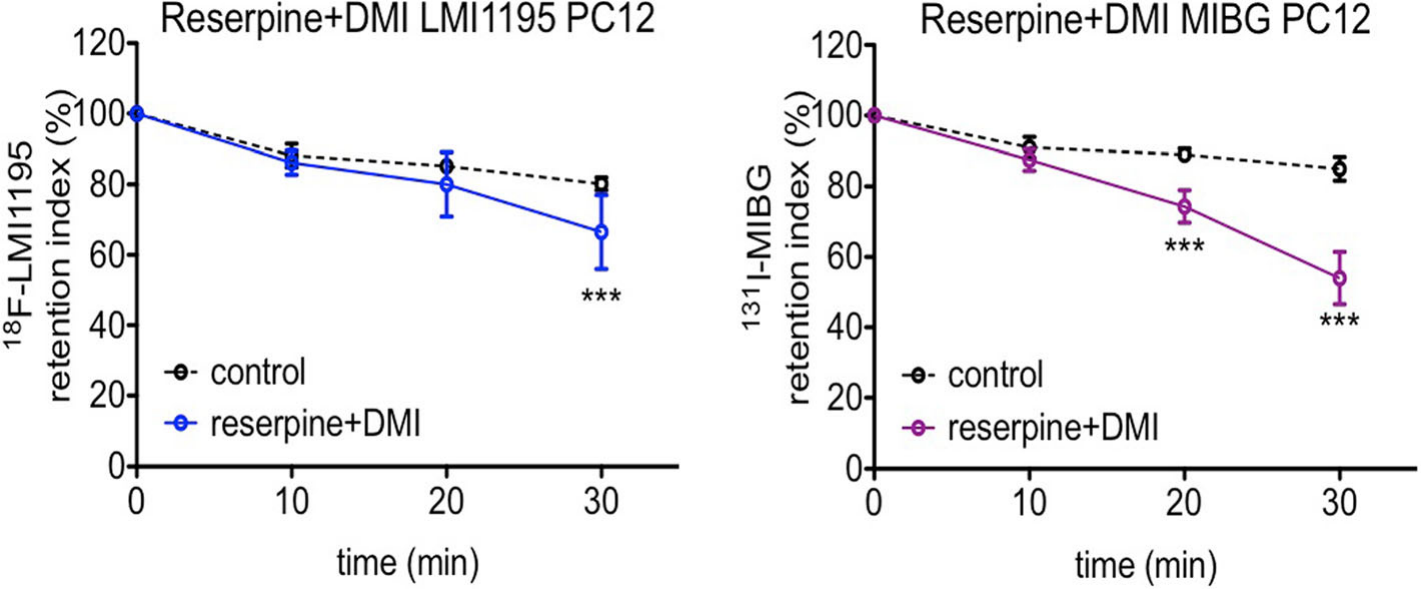
Co-exposing preloaded PC12 cells to reserpine and desipramine. The co-treatment also induced tracer release in the same mode as using reserpine alone. The release indices of either ^18^F-LMI1195 (left, *n* = 6 at each time point of both groups) or ^131^I-MIBG (right, *n* = 6 at each time point of both groups) reached significant differences compared to controls after 30 min. ***p < 0.001 vs. control group at the same time point; all data points presented as mean ± SD

The release of ^18^F-LMI1195 from PC12 cells by exposure to reserpine was time-dependent and reached significant differences after 30 min of treatment. This result is in accordance with our findings for ^131^I-MIBG (Fig. 3a). LMI1195 uptake reached a significant difference (*p* < 0.001) after 30 min of reserpine exposure with only 68 ± 3% left (vs. 80 ± 2% in controls). A similar pattern of tracer kinetics was confirmed using ^131^I-MIBG after reserpine exposure with retention of 65 ± 7% for the reserpine-treated group versus 85 ± 3% for the control group (*p* < 0.001). Applying the same protocol on SK-N-SH cells (vesicle-poor), 88 ± 2% retention of ^18^F-LMI1195 and 87 ± 2% of ^131^I-MIBG in the cells were recorded. However, no statistical difference was reached either for ^18^F-LMI1195 or for ^131^I-MIBG (Fig. 3b). Co-exposing the preloaded PC12 cells to both reserpine and the NET blocker desipramine, the tracer release showed a similar pattern to using reserpine alone with significant differences after 30 min of treatment (Fig. 3a vs. Fig. 4).

In summary, high concentration KCl and reserpine could enhance ^18^F-LMI119 washout from storage vesicle-rich PC12 cells. This washout as quantified as tracer releasing index could reach a significant difference after 30 min of treatment. In contrast, such effect could not be observed while using vesicle-poor SK-N-SH cells. As a golden reference, similar kinetics after KCl or reserpine treatment were also achieved using ^131^I-MIBG in the same cell lines. Furthermore, high concentration KCl exposure-induced tracer release was Ca^2+^ dependent as confirmed by suppressing the effect using calcium chelator EDTA and Ca^2+^-free buffer.

## Discussion

Several tracers sharing similarities in their benzylguanidine structure were designed to compensate for the disadvantages of the clinically used SPECT tracer MIBG. They all represent similarities to MIBG in order to achieve comparable in vitro intracellular retention and in vivo distribution properties [14]. Among them, ^18^F-LMI1195 is so far the best examined ^18^F-labeled PET tracer and has successfully proceeded with a clinical phase I trial [5]. In addition to the current literatures [5, 8, 9, 15], our research group has also performed a number of investigations with ^18^F-LMI1195 using animal models and ex vivo systems [11, 16, 17]. A further understanding of the properties of ^18^F-LMI1195 and its performance at a subcellular and molecular level is still of importance for its clinical application.

Therefore, we investigated the storage mechanism and depletion kinetics of LMI1195 on both rat pheochromocytoma PC12 and human neuroblastoma SK-N-SH cells, using ^131^I-MIBG as a comparator. The former cell line is rich of storage vesicles that could retain either the physiological neurotransmitter norepinephrine or radioactive tracers with analogous structures, whereas the SK-N-SH cells are poor of such secretory vesicles, and therefore, the taken-up tracers can only be stored in cytoplasm or mitochondria [18]. All cells were first preloaded with both tracers to reach equilibrium and thereafter were treated with either high concentration KCl buffer or reserpine in order to trigger the depletion of preloaded radiotracers.

As shown in Fig. 1, depolarization of PC12 cells caused by stimulation of high concentration KCl buffer evoked apparent tracer release, with approximately 60–70% depletion of additional ^18^F-LMI1195 or ^131^I-MIBG from the cells. By applying high concentration KCl to neuronal cells, Blaustein has proposed that neurotransmitter release from the nerve terminal is caused by Ca^2+^ influx via voltage-gated calcium channels [19]. Therefore, when using either Ca^2+^-free KCl buffer with Ca^2+^ chelator ethylene glycol-bis(2-aminoethylether)-*N,N,N′,N′*-tetraacetic acid (EGTA) or calcium channel blocker nifedipine, Araujo et al. further verified the suppression of norepinephrine release [20]. Similar conclusions were also drawn by Mandela et al. yielding that norepinephrine depletion is dependent on extracellular Ca^2+^ and could be fully suppressed by EDTA [21]. Thus, as expected, the outcome of exposing cells to Ca^2+^-free high KCl buffer containing EDTA lead to comparable findings in our study with a diminished release effect (Fig. 2).

This result attained from high KCl induction is consistent with the conclusion achieved from our research group using isolated rabbit hearts, in which the electrical provocation evoked enhanced tracer release [11]. Electrical field stimulation is known to induce norepinephrine overflow by releasing storage vesicles [22]. Since we could measure the radioactivity in the whole heart, including neuronal cells and myocytes, it was suggested that ^18^F-LMI1195 was taken up by the cells and stored within the vesicles [11]. In addition to our previous findings, we further confirmed this distinct uptake, storage, and release characteristics by using an in vitro assay.

As a human neuroblastoma cell line, SK-N-SH also expresses NET on the plasma membrane [23] and they are able to transport either ^131^I-MIBG or ^18^F-LMI1195 into cells. However, due to the shortage of storage vesicles, no apparent release of stored tracers could be observed after the application of high KCl buffer compared to controls (Fig. 1b). The response of high KCl-leading tracer release compared with the control group is of utmost importance: Since no statistical difference could be observed between both groups, a robust conclusion can be derived from the setup of our experiment.

**Fig. 5:**
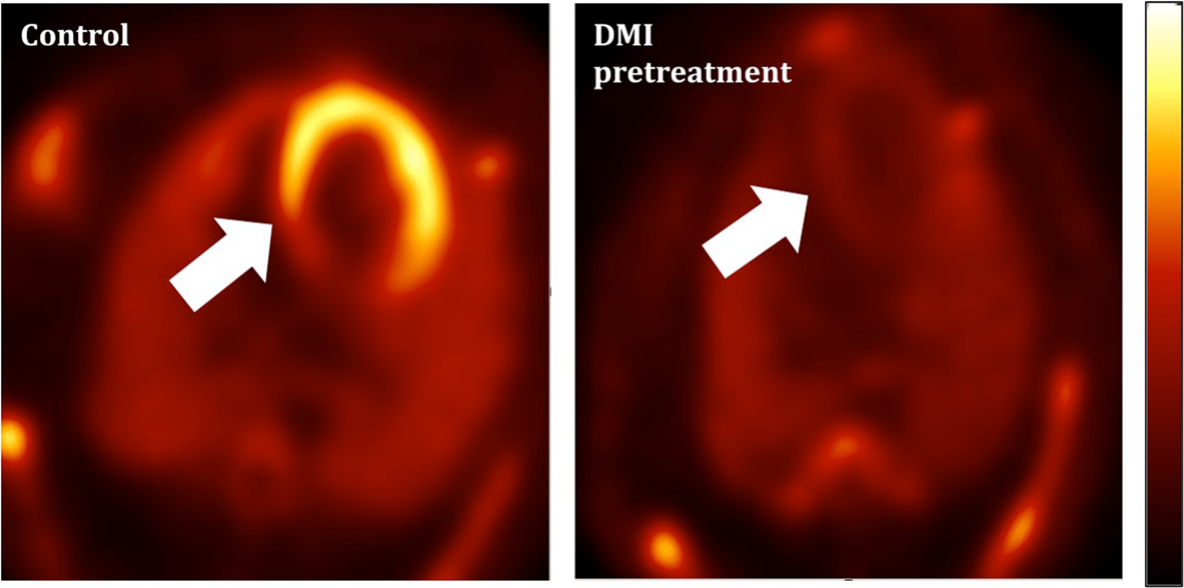
Transverse image of ^18^F-LMI1195 uptake in rabbit heart, showing control (left) and with DMI pretreatment (right). Averaged scan 10–30 min after tracer injection. With permission of [16]

**Fig. 6:**
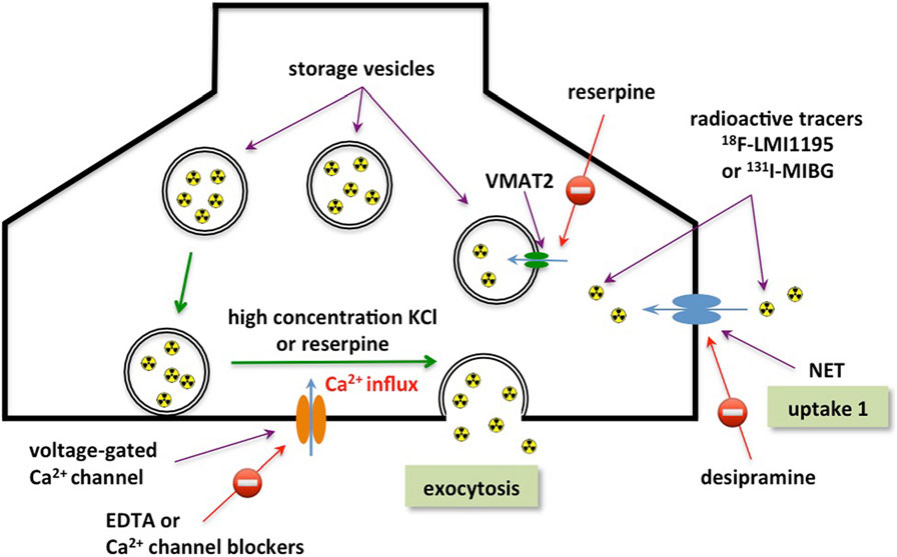
Illustration of radiotracer uptake, storage, and release mechanisms in PC12 cells. In PC12 cells, radiotracers (^18^F-LMI1195 or ^131^I-MIBG) that have been selectively taken up into the cells are first stored in storage vesicles and can be released by either high concentrations of KCl or reserpine. This procedure is Ca^2+^ dependent

Reserpine is known for its potential to release norepinephrine from synaptic nerve cells by triggering the exocytosis of storage vesicles [21]. In this study, reserpine induced significant tracer release after 30 min of its application to vesicle-rich PC12 cells (Fig. 3), whereas such an effect was not observed in SK-N-SH cells, which is in accordance with the conclusion drawn by Smets et al. from a reserpine-induced MIBG depletion study [24]. Due to the deficiency of storage vesicles in SK-N-SH cells, no clear tracer overflow, either with ^18^F-LMI1195 or ^131^I-MIBG, could be observed. The efflux of tracers from SK-N-SH cells may be only due to slow passive diffusion. The current study of using either high concentration KCl or reserpine is the opposite way as the results achieved from rabbit heart [16], in which pretreatment of desipramine was followed by tracer injection (Fig. 5). Firstly, this in vivo study provided the first proof of successfully prohibiting the uptake of tracer into storage vesicles by using desipramine. Secondly, the in vitro cell study demonstrated the clear depletion mechanism of an already taken-up tracer in the storage vesicles. By comparing the two methods (high concentration KCl and reserpine), it was revealed that the application of these exogenous radioactive sympathetic nerve tracers apparently mimics the physiological neurotransmitter norepinephrine turnover quite well, including transporter-mediated uptake as well as modes of storage and exocytosis (Fig. 6). Integrating our previous animal study (Fig. 5) and ex vivo results [11, 16, 17] with the present in vitro findings, the intracellular behavior of ^18^F-LMI1195 is analogous to its SPECT counterpart MIBG and the neurotransmitter norepinephrine.

Similar to high KCl-induced exocytosis, reserpine-mediated ^18^F-LMI1195 release is also Ca^2+^ dependent. Mandela et al. have investigated and reported how reserpine influences NET in a non-competitive manner by Ca^2+^ dependency and how it interferes with the interaction between NET and norepinephrine storage vesicles. Strikingly, it was revealed that reserpine induces a non-competitive inhibition of norepinephrine uptake in PC12 cells [13]. This effect requires the presence of vesicular monoamine transporter (VMAT) and storage/secretory vesicles, which explains the finding for exposure to reserpine alone and reserpine/desipramine-induced tracer release—a demonstration of analogous uptake and efflux mechanisms associated with the benzylguanidine structure common to both tracers (Fig. 4). By contrast, as demonstrated previously, cardiac retention of ^11^C-hydroxyephedrine (^11^C-HED) is mediated through a continuous cyclical mode of release (diffusion out) and reuptake via NET from the nerve terminal [11, 16]. ^11^C-HED showed enhanced washout from both in vivo and isolated perfused rabbit heart after desipramine chase. On the other hand, ^18^F-LMI1195 and MIBG are not sensitive to a NET inhibitor chase protocol in an in vivo setting, which was imitated in the present in vitro study by adding desipramine while incubating with reserpine (Fig. 4). Therefore, on a subcellular level, a stable vesicle-storing mechanism mimicking physiological norepinephrine turnover was corroborated.

It should be mentioned that in addition to the application of these NET tracers in cardiac diseases, there are many potential applications in tumor diagnosis [25]. ^123^I-MIBG imaging had been used in the evaluation of neuroblastoma for years [26]. ^18^F-LMI1195 would also be available because of their structural and property similarities in NET imaging: A previous study of high and specific accumulation of LMI1195 in pheochromocytomas has already made the first attempt in proving this potential [15].

## Conclusions

Our study demonstrated the subcellular and molecular uptake and release mechanism of the novel sympathetic nerve PET tracer ^18^F-LMI1195. These findings are analogous to findings for the structurally related and widely used SPECT predecessor MIBG. Both high concentration KCl and reserpine induce the depletion of ^18^F-LMI1195. The proposed mechanism of vesicle storage and release is consistent with the conclusions suggested from previous studies using both ex vivo isolated perfused and in vivo rabbit hearts. To sum up, we herein demonstrated that ^18^F-LMI1195 is a promising tracer for visualizing the cardiac innervation by mimicking the physiologic neurotransmitter norepinephrine. It can provide similar properties as MIBG in a clinical setting along with the advantages of ^18^F-labeling and PET imaging technology.

## Additional file

Additional file 1: Preparation of buffer systems. (DOCX 107 kb)
