# Supplementary material for: Subcellular storage and release mode of the novel 18F-labeled sympathetic nerve PET tracer LMI1195
Source: EJNMMI Res. 2018 Feb 6;8:12. doi: 10.1186/s13550-018-0365-9 (PMC5801140; doi:10.1186/s13550-018-0365-9)
Supplement: Additional file 1: — Preparation of buffer systems. (DOCX 107 kb) [file 13550_2018_365_MOESM1_ESM.docx]

**Supporting Information**

**Preparation of buffer systems:**

HBS buffer (135 mM NaCl, 5 mM KCl, 0.6 mM MgSO_4_, 2.5 mM CaCl_2_, 10 mM HEPES, 6 mM D-glucose, 0.2 mM ascorbic acid)

High KCl buffer (40 mM NaCl, 100 mM KCl, 0.6 mM MgSO_4_, 2.5 mM CaCl_2_, 10 mM HEPES, 6 mM D-glucose, 0.2 mM ascorbic acid)

HBS Ca^2+^ free buffer (135 mM NaCl, 5 mM KCl, 0.6 mM MgSO_4_, 10 mM HEPES, 6 mM D-glucose, 0.2 mM ascorbic acid, 1 mM EDTA)

High KCl Ca^2+^ free buffer (40 mM NaCl, 100 mM KCl, 0.6 mM MgSO_4_, 10 mM HEPES, 6 mM D-glucose, 0.2 mM ascorbic acid, 1 mM EDTA)

**Protein concentration testing for high concentration of KCl treatment:**

High KCl on PC12 cells:

| **Conditions** | **Protein concentration** | **Average** | **SD** | ***t*-test** | ***p*-value** |
| --- | --- | --- | --- | --- | --- |
| HBS 30min | 289.7 | 268.067 | 21.502 | 0.118 | >0.1 |
| HBS 30min | 267.7 |  |  |  |  |
| HBS 30min | 246.7 |  |  |  |  |
| High-KCl 30min | 247.1 | 243.733 | 9.452 |  |  |
| High-KCl 30min | 251.1 |  |  |  |  |
| High-KCl 30min | 233.1 |  |  |  |  |
| EDTA HBS 30min | 110.1 | 118.178 | 9.131 |  |  |
| EDTA HBS 30min | 116.4 |  |  |  |  |
| EDTA HBS 30min | 128.1 |  |  |  |  |
| EDTA High-KCl 30min | 96.7 | 92.400 | 9.905 |  |  |
| EDTA High-KCl 30min | 81.1 |  |  |  |  |
| EDTA High-KCl 30min | 99.4 |  |  |  |  |
| HBS 20min | 206.1 | 208.067 | 3.756 | 0.407 | >0.1 |
| HBS 20min | 205.7 |  |  |  |  |
| HBS 20min | 212.4 |  |  |  |  |
| High-KCl 20min | 217.4 | 216.067 | 9.735 |  |  |
| High-KCl 20min | 225.1 |  |  |  |  |
| High-KCl 20min | 205.7 |  |  |  |  |
| HBS 10min | 215.1 | 214.400 | 9.684 | 0.582 | >0.1 |
| HBS 10min | 204.4 |  |  |  |  |
| HBS 10min | 223.7 |  |  |  |  |
| High-KCl 10min | 259.4 | 227.733 | 30.567 |  |  |
| High-KCl 10min | 225.4 |  |  |  |  |
| High-KCl 10min | 198.4 |  |  |  |  |

High KCl on SK-N-SH cells:

| **Conditions** | **Protein concentration** | **Average** | **SD** | ***t*-test** | ***p*-value** |
| --- | --- | --- | --- | --- | --- |
| HBS 30min | 15.2 | 21.313 | 5.590 | 0.304 | >0.1 |
| HBS 30min | 26.1 |  |  |  |  |
| HBS 30min | 22.7 |  |  |  |  |
| High-KCl 30min | 28.5 | 27.475 | 3.154 |  |  |
| High-KCl 30min | 23.9 |  |  |  |  |
| High-KCl 30min | 30.0 |  |  |  |  |
| EDTA HBS 30min | 10.3 | 16.263 | 5.216 |  |  |
| EDTA HBS 30min | 20.0 |  |  |  |  |
| EDTA HBS 30min | 18.5 |  |  |  |  |
| EDTA High-KCl 30min | 2.4 | 0.202 | 2.018 |  |  |
| EDTA High-KCl 30min | -0.3 |  |  |  |  |
| EDTA High-KCl 30min | -1.5 |  |  |  |  |
| HBS 20min | 27.6 | 29.899 | 3.035 | 0.237 | >0.1 |
| HBS 20min | 33.3 |  |  |  |  |
| HBS 20min | 28.8 |  |  |  |  |
| High-KCl 20min | 34.5 | 36.061 | 4.296 |  |  |
| High-KCl 20min | 32.7 |  |  |  |  |
| High-KCl 20min | 40.9 |  |  |  |  |
| HBS 10min | 17.9 | 16.263 | 1.432 | 0.150 | >0.1 |
| HBS 10min | 15.2 |  |  |  |  |
| HBS 10min | 15.8 |  |  |  |  |
| High-KCl 10min | 9.1 | 11.414 | 2.613 |  |  |
| High-KCl 10min | 10.9 |  |  |  |  |
| High-KCl 10min | 14.2 |  |  |  |  |
